# When It Strikes, Are We Ready? Lessons Identified at the 7th Planetary Defense Conference in Preparing for a Near-Earth Object Impact Scenario

**DOI:** 10.1007/s13753-021-00389-9

**Published:** 2022-01-07

**Authors:** Shirish Ravan, Tom De Groeve, Lara Mani, Einar Bjorgo, Richard Moissl, Jose Miguel Roncero, Katherine Rowan, David Schuld, Leviticus A. Lewis, Romana Kofler

**Affiliations:** 1 United Nations Office for Outer Space Affairs (UNOOSA), United Nations, 1220 Vienna, Austria; 2 European Commission Joint Research Centre, 21027 Ispra, Italy; 3 Centre for the Study of Existential Risk, University of Cambridge, Cambridge, CB2 1SB UK; 4 Division for Satellite Analysis, United Nations Institute for Training and Research (UNITAR), 1202 Geneva, Switzerland; 5 European Space Agency/European Space Research and Technology Centre (ESA/ESTEC), 2201 AZ Noordwijk, The Netherlands; 6 Security and Situational Awareness Unit, European Commission’s Directorate-General for European Civil Protection and Humanitarian Aid Operations, 1049 Brussels, Belgium; 7 Department of Communication, George Mason University, Fairfax, VA 22030 USA; 8 Hagerty Consulting, Evanston, IL 60201 USA; 9 Response Operations Division, Federal Emergency Management Agency, Washington, DC, 20024 USA

**Keywords:** Asteroid threats, Disaster risk reduction, Planetary defense, Risk management and prevention

## Abstract

Near-Earth object (NEO) impact is one of the examples of high impact and low probability (HILP) event, same as the Covid-19 pandemic the world faces since the beginning of 2020. The 7th Planetary Defense Conference held by the International Academy of Astronautics (IAA) in April 2021 included an exercise on a hypothetical NEO impact event, allowing the planetary defense community to discuss potential responses. Over the span of the 4-day conference this exercise connected disaster response and management professionals to participate in a series of panels, providing feedback and perspective on the unfolding crisis scenario. The hypothetical but realistic asteroid threat scenario illustrated how such a short-warning threat might evolve. The scenario utilized during the conference indicates a need to prepare now for what might come in the future, because even with advance notice, preparation time might be minimal. This scenario chose Europe for the impact, which may likely cope with such a disaster, through the Union Civil Protection Mechanism (UCPM) and other solidarity and support mechanisms within the European Union (EU), as well as with potential support from international partners. This short article raises concern about other areas in the world on how they may access NEO impact information and cope with such disasters. It also provides an idea on vast scale of such disaster vis-à-vis the current capacity of response systems to cope with a larger event in Europe or elsewhere. This scenario showed that planetary defense is a global endeavor. Constant engagement of the planetary defense and disaster response communities is essential in order to keep the world safe from potential disasters caused by NEO impacts.

## Realizing the Reality of High Impact and Low Probability Events: From Pandemics to Near-Earth Object Impacts

The world is experiencing a once in a century pandemic event. The memories of past pandemics, such as the 1918 Spanish Flu that is estimated to have killed over 50 million people from 1918 to 1920 (Taubenberger and Morens 2006), faded in due course, and lessons identified and learned hard were forgotten. Although countries around the world are now much more equipped with more advanced knowledge and technologies, the world is still grappling with the Covid-19 pandemic and dealing with the collateral effects of the pandemic including economic recession and long-term health impacts. The pandemic is one example of a high impact and low probability (HILP) event.

Another such potential HILP event may be a near-Earth object (NEO) impact, such as the impact of an asteroid striking somewhere around the globe. Although impacts from large NEOs (> 300 m) are very rare (once every 70,000 years or more), the risk of impact from objects as small as 15−20 m is much more likely with approximately one impact every 100 years (NASA 2014). Impacts from objects of even a smaller size can cause serious damage and have long-term consequences if they hit populated areas. Today, in order to increase resilience to such threats, scientific communities are using more advanced telescopes to find and surveil asteroids and comets that might be a threat. If one is found, the goal would be to deflect or disrupt the object before impact—an effort that requires several years of advanced detection and warning.

Disaster management organizations have been developed and evolved to deal with disaster incidents such as floods, droughts, hurricanes, earthquakes, landslides, snow avalanches, and oil spills. For many organizations and their respective host nations, a HILP event like a NEO impact has not been part of their traditional disaster preparedness and response strategy. The Sendai Framework for Disaster Risk Reduction 2015−2030 (UNISDR 2015)—the key global framework endorsed by the United Nations member states for building disaster resilience—does not mention HILP events. Lessons learned from the Covid-19 pandemic have been an eye opener for the disaster management community and a reminder that there is a need to prepare for HILP events and practice preparedness and response through hypothetical scenarios.

## Exercise Scenario: The Threat Identified and Preparing for Impact

The 7th Planetary Defense Conference (PDC) held by the International Academy of Astronautics (IAA) on 26−30 April 2021 included a scenario exercise of a hypothetical NEO impact event, and practitioners across the field of planetary defense discussed what it would take to respond. The biannual conference—hosted by the United Nations Office for Outer Space Affairs (UNOOSA), in cooperation with the European Space Agency (ESA)—brought together experts involved in monitoring NEOs, from fields such as planetary defense, disaster risk reduction, astronomy, international law, and astronautics, to discuss the threat posed to the Earth by asteroids and comets, and decide on actions that might be taken to deflect a threatening object. Ten disaster response and management professionals, from a range of institutions^1^ who are normally involved in preparing for natural hazards and disasters such as earthquakes, floods, severe storms, volcanic eruptions, tsunamis, wildfires, explosions, and landslides participated in a series of four panels^2^ to provide critical feedback and perspective on a hypothetical crisis scenario.

The 4-day conference exercise used a hypothetical but realistic asteroid threat scenario to illustrate how such a short-warning threat might evolve over the span of a 4-day period:

- Day 1: An object has been discovered that might hit anywhere on the Earth in about 6 months. Presentations were made to describe what is known about the object, the likelihood that impact will occur, and the damage that might occur should it impact.
- Day 2: Experts discussed possibilities for deflecting or disrupting the object.
- Day 3: Impact is certain and will occur within a large geographic region that includes several nations. Experts provided detailed summaries of potential impact consequences.
- Days 3 and 4: A panel of disaster managers discussed actions to be taken to prepare and protect the public.

**Fig. 1:**
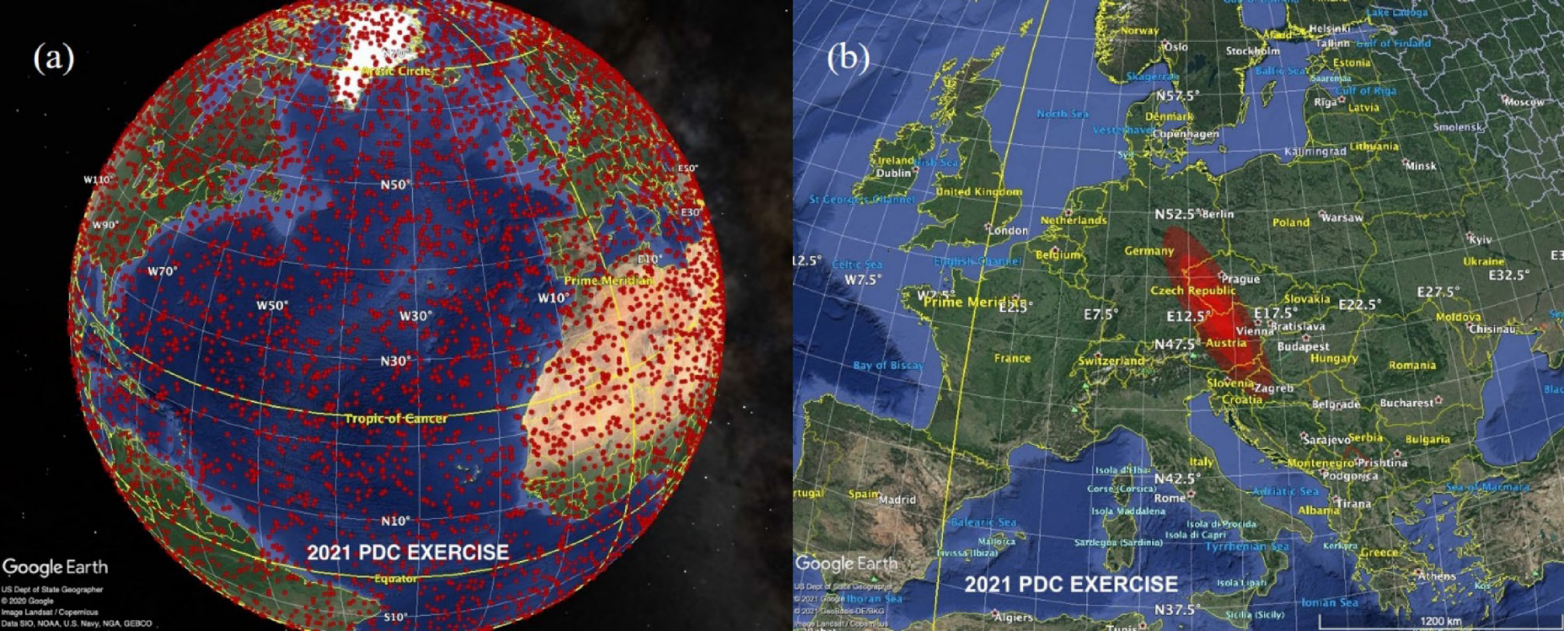
Maps taken from the hypothetical scenario exercise for a near-Earth object impact “2021 PDC Exercise” during the 2021 7th Planetary Defense Conference (PDC) held by the International Academy of Astronautics (IAA). **a** The map shows the initial estimates for impact locations (red dots) based on early modeling during the scenario exercise; **b** map of the final expected impact corridor as constructed by continued modeling during the scenario exercise. *Source* https://cneos.jpl.nasa.gov/pd/cs/pdc21/

Figure 1 shows the potential impact location as it progressed through the scenario exercise, culminating with the final expected impact corridor extending across the Germany, Austria, and Czech Republic border region.

## Opportunity Identified: The Need to Bridge Planetary Defense and Disaster Management

Exercises like the one in the 7th Planetary Defense Conference are essential in stress-testing response mechanisms and highlighting points of weakness. Global risks such as a NEO threat require the combination of many disciplines and actors such as political actors, disaster risk managers, nongovernmental organizations, academics, and community members. A holistic approach to such exercises ensures all voices and values are considered, while also adding realism to the scenarios.

The hypothetical scenario of a NEO being discovered and its probable impact being a few months or less away raised the following questions for disaster management specialists:

- Are we prepared to provide accurate information to the public about such an event?
- If the impact area were to involve several nations, how will their disaster management organizations coordinate responses?
- Can large populations be evacuated quickly from an area where impact would end all life and destroy all infrastructure?
- Is there relevant experience from planning for and responses to volcanic eruptions, hurricanes, earthquakes, tsunamis, and other natural hazards and disasters?
- Is there relevant experience from disasters at critical infrastructure locations (for example, nuclear power plants, chemical plants, oil/gas facilities)?
- How has the public reacted in the case of past disasters?

The exercise discussions that took place during the conference brought together a panel of disaster management specialists to deal with these questions. They also offered disaster response specialists the opportunity to become more acquainted with disasters and risks posed by asteroids and comets as natural hazards. Lastly, the discussions took into account lessons learned from building resilience, providing early warning, and offering responses to other disasters, and their perspective was provided with a focus on HILP disasters such as a NEO impact and the Covid-19 pandemic.

## Preparedness Lessons Learned from the 7th Planetary Defense Conference

Through a series of expert discussions, the critical nature of applying principles of disaster management to a NEO impact was apparent. Key points and considerations to further action from the perspective of disaster management are discussed in this section.

### Detection and Early Warning

Large asteroid impacts are a global threat and the right early warning information, its effective dissemination, and positive messages are powerful tools to counter irrational responses and panic.

#### Large Asteroid Impacts Are a Global Threat, Always, and Observation Networks and International Collaboration are Important

Due to the difficulties of determining precise asteroid orbits, it is often difficult to precisely predict the impact location ahead of time. Through well-established observation networks and international collaboration, the time needed to reduce the many uncertainties of the impact parameters can often be shortened significantly. But even when an impact location is known for large events, dealing with the damage can exceed the capacity of individual nation states. Therefore, a broad international and interagency collaboration among disaster mitigation actors strengthens the overall resilience against impact threats.

#### The Right Information Needs to be Disseminated to the Right People at the Right Time

One of the big lessons learned from the impact event exercises is that the flow of information is a key element of enabling adequate responses. Therefore, the development of fast and reliable information exchange must continue in order to keep all relevant actors (including the media and the public) up-to-date on the latest developments.

#### Positive Messages are a Powerful Tool to Counter Irrational Responses and Panic

Experience from many past disasters has taught us that positive messages are a vital element in helping to unite people(s) in the face of adversity. The knowledge and confidence of being able to mitigate the impact of a disaster can greatly boost the morale of the affected population and the effectiveness of the helpers. Tools to detect and manage misinformation are also crucial to avoid uncontrolled development of misleading news stories.

#### Early Warning will be Effective if the Countries Know About Underlying Vulnerabilities

Knowing about underlying risks, for example critical but vulnerable infrastructure such as nuclear facilities, transport lines, and dams, and taking appropriate measures to protect against and mitigate a NEO impact is important and calls for integrating early warning information with risk information. Regularly updated national risk assessments—common practice in the European Union Civil Protection Mechanism—are a useful practice (Poljansek 2021).

#### Early Warning Needs to be Translated into Early Planning and Early Action

While early warning can enable the transmission of relevant information to relevant people at the right time, early warning must be translated into early planning and, if needed, early action. The scientific community plays an essential role in enabling this process by providing fact-based information to decision makers. Analytical cells or teams in disaster response organizations can translate scientific information into operational or actionable information, that is, into language that decision makers fully understand.

### Advance Planning and Exercise

Events like NEO impacts need holistic, comprehensive, and targeted approaches and multiagency involvement to disaster preparedness, supported by long-term thinking and civil protection codes.

#### The Time for Preparation is Now

This scenario offers some time for preparedness, but would a few months be enough? A holistic and comprehensive approach to disaster preparedness needs to take this and other scenarios into account. Such an approach would result in a multifaceted approach in terms of building capacities and capabilities for disaster management. For example, what might be useful for a large volcanic eruption or a large tsunami might also be used for an asteroid impact. The experience that civil protection authorities and organizations have gained in responding to earthly natural hazards and disasters can very well be applied to a NEO disaster.

Through impact event simulations and exercises, the strain on resources in pre-impact preparation and mitigation became clear. The large uncertainties that planetary defense professionals and emergency managers were confronted with complicate the process even further. Through regular research activities and controlled experiments—that is the Double Asteroid Redirect Test (DART)/Hera Mission (ESA n.d.a)—critical knowledge can be acquired, which will help to deal more efficiently with actively defending ourselves against hazardous objects. Furthermore, regular international and multiagency training and exercises of different scenarios will help to educate and prepare disaster managers across the planet.

#### Better Assessment is Needed for Targeted Preparedness

The scenario offered some time for more targeted preparedness, for example, to prepare evacuation and response plans and mobilize assets to support response operations. However, the initial impact area assessment is not good enough to enable targeted preparedness (for example, an area 100 × 1000 km is too large for an evacuation, if the final impact is going to cover a 10 × 10 km area). Until better assessments are available, there will be a need to handle public communication and, most importantly, control possible panic. In addition, measures will need to be taken in terms of possible mis- and disinformation, including event deniers or people simply unwilling to evacuate, as well as with extreme cases such as adrenaline junkies, disaster chasers, and doomsday tourists. Reconstruction plans would also be paramount, in particular to support populations being evacuated from the impact area (once that area has been narrowed). Reconstruction plans, if announced before impact, can ease the desperation that most evacuees are likely to experience. They must know they are not alone.

#### Existing Networks, Tools, and Technologies Should be Leveraged for Better Preparedness

In order to ensure preparedness, it is important to engage with existing networks. One such network is the Global Disaster Alert and Coordination System (GDACS), a joint European Commission and United Nations initiative that serves the international disaster community and national entities with relevant alerts and coordination tools. One of these tools is the Virtual On-Site Operations Coordination Centre (Virtual OSOCC). This tool is a live coordination platform during disasters, but also a tool for preparedness exercises at the international level. The PDC community could use this platform to engage with various relevant actors.

Bringing NEO scenarios into the dialogues of existing international science-policy networks, such as the European Commission’s Disaster Risk Management Knowledge Centre, is an effective way to mainstream such scenarios in an all-hazard and all-sector preparedness approach, involving all Directorate Generals (Casajus Valles et al. 2021). The new Knowledge Network of the European Union’s Civil Protection Mechanism similarly would engage with all member states and help put this “new” risk on the radar of governments.

In terms of satellite imagery assessment of an affected area, entities such as the UN Institute for Training and Research (UNITAR) Operational Satellite Applications Programme (UNOSAT) and the United Nations Platform for Space-based Information for Disaster Management Emergency Response (UN-SPIDER), along with mechanisms like the International Charter Space and Major Disasters, the Copernicus Programme, in particular its Emergency Management Service, and the International Network for Multi-Hazard Early Warning Systems (IN-MHEWS) could quickly estimate the impact, in terms of damage to buildings and infrastructure.

#### Preparedness Should Include a Multiagency Approach

Whether an active threat event, a natural hazard-related disaster, or a pandemic—disasters have taught communities that plans, training, and exercises must consider a multiagency response and recovery effort. No issue is siloed exclusively to a single responding organization, especially in planetary defense (imagine the scale of mutual aid in preparing for, responding to, and recovering from a NEO impact). As space agencies and partner organizations collaborate globally, so too must the planetary defense community reflect on how they will engage with national and local agencies in preparing for a NEO impact. In this scenario, the spectrum of coordinating organizations (local to global) will be an immense undertaking: evacuation, mitigating the impact to critical energy infrastructure, addressing the refugee status of evacuees who know that their community may no longer exist, search and rescue operations post-impact, and community resilience all bring unique agencies to the preparedness table.

### Social, Economic, and Political Considerations

Months or years of early warnings, and uncertainty associated with impact location of a NEO impact may cause political or social unrest and fatigue. Tried and tested disaster management systems and protocols need to be flexible to deal with these social-economic-political tides.

#### Be Aware of Influences that May Steer Disaster Management

When planning for disaster preparedness, secondary and tertiary issues including political and social influences need to be considered by disaster managers. Tried and tested disaster management systems and protocols need to be flexible and adaptable in their application. Traditional or planned protocols may be challenging or impossible to implement if the political or social tides against them are too strong. COVID-19 response protocols ranging from quarantine and isolation to vaccine point of dispensing have needed to adapt to what pre-COVID-19 pandemic plans disaster management agencies assumed (massive vaccine point of dispensing with open supply and large demand). A key opportunity for improvement learned from the management of COVID-19 is to consider how to develop resilient plans and maintain situational awareness of the policy-level leadership with respect to their existence and the methods to utilize them.

#### Be Prepared for Responder Fatigue in a Slow-Burning Disaster

Slow-forming and long-lasting disasters (like a global pandemic) have illustrated how these events can fatigue responders, decision makers, and the public. A NEO impact may be known months or years in advance of its actual target impact. Hysteria will be quelled, and initial alert and immediate desire to act will transform into weeks upon weeks of response. Countering fatigue (including providing mental health services to responders) needs to be considered in advance planning.

#### Creating a Forum of Discussion and Collaboration in Exercises is Critical

After initial reactions to a NEO impact, the consequences are catastrophic and real. Exercises—discussion-based, functional, game-based to full-scale—are opportunities to engage with challenging issues in a no-fault learning environment. While some may discount a NEO impact, the opportunity to interact with this scenario reveals gaps and way forward to strengthening partnerships and capabilities.

### Risk Communication

Risk communication must include building cultural sense through storytelling about risks and disasters, listening to vulnerable groups to address their concerns, working with journalists to counter false information and arranging communication between victims and communities that experienced disasters in the past.

#### Reliable Risk Communication Through Storytelling About Risks and Consequences Helps in Achieving Resilience

Research suggests that resilient communities achieve resilience in part by sharing stories about danger and disasters, and by seeking to confirm information (milling) through monitoring media and interpersonal talk. These communicative processes contribute to a sense of community, belonging, and efficacy against adversity (Spialek and Houston 2019). Features of effective communication were identified as resources that assist communities in surviving extreme events and restoring their ways of life. Communication best practices assist what the U.S. Federal Emergency Management Agency calls a culture of preparedness for the whole community, not just some (FEMA 2014).

#### Stories are Powerful Tools for Effective Risk Communication in all Cultures

“Reliable risk communication” underlines the importance of storytelling as stories are a universal form of human communication. All cultures generate and share them. Research on brain function indicates that stories engage both the primitive part of the brain, the amygdala, which responds quickly to danger, and the advanced part of the brain, the cortex, used to analyze and select protective action (Loewenstein et al. 2001). Stories may also foster a sense of belonging and community that supports community resilience. For example, Scherzer et al. (2019) studied Norwegian communities devastated by disasters such as rock slides. Some of these events were so painful that some community elders had never shared stories about them until they were interviewed. These scholars identified communicative practices and other features that distinguish high-resilience and high-vulnerability communities.

#### Working with Conscientious Journalists and “Pre-Bunking” May Counter False Information

At the 2021 Planetary Defense Conference, science writer Sarah Scoles discussed the importance of science agencies building good working relationships with reliable journalists. She also described techniques for helping stakeholders detect false information. One of those techniques is called “pre-bunking,” and research shows that pre-bunking is effective. Pre-bunking involves alerting audiences to the techniques that are being used to spread false information (Linden 2019). For example, one manipulative technique is that of “false equivalency.” It is a false equivalency, for example, to say the views of one person equal the findings of thousands of scientists and engineers whose analyses of a particular danger are vetted through peer review.

#### Equitable and Effective Early Warning Encourages Discussions and the Preparation of Protective Actions Among Vulnerable Groups

Research shows that people do not—cannot—follow instructions without thinking. Instead, when warned, they seek information or “mill” to confirm what they have heard. They talk to others around them to see what others plan to do in response to some warning or danger (Wood et al. 2018). This means that effective warnings must “activate” talk or “milling” among people about protective action. It is especially important to activate this talk among vulnerable groups.

#### Effective Warnings Develop from Research, Specifically from Listening Systematically to the Logistical Challenges of Vulnerable Groups Prior to Disasters

Evacuation may be one recommended protective action if a near-Earth object is heading toward the Earth. But evacuation can be difficult. This process would be difficult for farmers who have large animal herds, those who are hospitalized, the elderly, those who rarely travel, the low-resourced, and so forth. Best practices for thinking through these logistical challenges include: identifying types of vulnerability from prior disasters; and finding “community influencers” such as local religious or business leaders trusted by vulnerable groups and supporting these influencers in correcting mis- or disinformation as it emerges. An additional systematic approach to learning the logistical challenges and concerns of vulnerable groups is to conduct “deliberative meetings” where randomly selected participants are paid to attend day-long sessions to explain a particular danger, discuss options for its management, and, with peers, discuss protective actions that seem feasible for them and their community (Fishkin and Luskin 2005). Reports are written about findings from a deliberative meeting and sent to decision makers.

#### Communities Coping with the Destruction of Their Homeland May Find Support from Other Communities Who Faced Somewhat Similar Disastrous Events

Arranging communication between asteroid strike victims and victims of other disasters, such as volcano eruptions, may provide a helpful form of emotional and informational support to potential asteroid-strike victims. One barrier to evacuation is concern about financial and emotional losses (Grothmann and Reusswig 2006), and those who have faced similar losses may be able to offer guidance and emotional support.

### Governance

The principle of “leaving no one behind” calls for an inclusive effort of incorporating NEO preparedness and response in the governance at national, regional, and international levels.

#### Special Efforts are Needed to Introduce NEO Risk to Least Developed Countries (LDCs), Small Island Developing States (SIDS), and Landlocked Developing Countries (LLDCs)

Many developing countries, in particular LDCs, SIDS, and LLDCs, have not benefited as much as they could have from advances in the science, technology, and governance behind early warning systems. National disaster management strategies are guided by the four priorities and seven targets of the Sendai Framework. Community disaster management authorities that are engaged in implementing the Sendai Framework should be made aware of the NEO threat through various national, regional, and international platforms. This is the way to inform disaster management agencies of all countries and promote inclusion of NEO risks in national and regional disaster management strategies, which will avoid a panic situation in case of disaster striking.

It is important that national, regional, and intergovernmental disaster management organizations and space agencies engage with the International Asteroid Warning Network (IAWN) (UNOOSA n.d.a) and the Space Mission Planning Advisory Group (SMPAG) (UNOOSA n.d.b) to contribute to monitoring asteroids in the same way they are engaging in dealing with disasters like floods, droughts, storms, earthquakes, and so on.

#### Long-Term Thinking is Required to Ensure a Robust and Secure Approach

Lack of long-term thinking in governments and international organizations can hinder the preparation and mitigation capabilities against high-impact low-probability events. For example, the approach to the climate crisis is a particularly relevant case in the United States, where changes in administration have seen polarized approaches to the mitigation of the risks. A potential solution is the adoption of a strategic position within governmental structures to conduct depoliticized risk assessments and to hold politicians accountable for failing to act responsibly on risk.

#### Civil Protection Authorities Play a Critical Role

In this scenario, the involvement of civil protection authorities would be essential. Civil protection is the backbone of emergency preparedness and response in many countries, and in particular in the countries affected in the scenario. Through their experience in disaster management, and taking advantage of their coordination and resilience ability, civil protection authorities are very likely to be tasked to carry out many of the pre- and post-event actions. Their role would be central as well as essential.

International coordination and cooperation of civil protection preparedness and response activities in view of a NEO impact would be essential. The scenario impact area in Europe helps showcase what could be considered an international and supranational best practice for disaster management, the Union Civil Protection Mechanism (UCPM) of the European Union. Composed of the 27 EU member states as well as six participating states (Iceland, Norway, Serbia, Montenegro, North Macedonia, and Turkey), the UCPM pulls together civil protection and emergency response teams and assets from all over Europe to respond to disasters globally. At the heart of the organization is the Emergency Response Coordination Centre (ERCC), which monitors events and coordinates the delivery of assistance provided through the UCPM. The UCPM also relies on the rescEU, an EU-level layer of emergency response assets meant to complement national response capacities (ESA n.d.b). With over 400 activations worldwide since 2013, the UCPM is a successful example of international cooperation and solidarity with respect to responding to disasters.

#### Secure Funding is Needed to Tackle HILP Risks

Long-term financial commitments are required in order to secure the continued efforts to increase resilience towards NEO impacts. Head of the ESA’s Planetary Defence Office and member of the planetary defense conference organization committee Detlef Koschny said, on reflection of the lessons learned from the conference, “Simply thinking in annual or biennial planning cycles, which is how many budgets at public institutions are set, is not good enough to address a risk that has been hundreds of millions of years in the making” (ESA 2021).

### Outreach and Education

Public outreach is fundamental to avoiding panic and ensuring well-planned evacuations. This must be done in close collaboration with national entities (Wood et al. 2018).

#### Do Not Underestimate the “Power of Publics”

The public should be considered a plurality of heterogeneous “publics” of different beliefs, values, languages, and priorities. During the COVID-19 pandemic, the level of compliance of the public has been incredibly high in the face of uncertainty. Compliance with draconian lockdowns, curfews, and health protection measures, such as wearing masks and physical distancing, have seen wide acceptance, evidenced by the reduction in transmission rates and loss of life. Additionally, altruistic behaviors have been observed and have prevailed throughout the pandemic, with communities working together in the face of uncertainty.

#### The Importance of Education and Outreach Highlighted in the Sendai Framework is Valid for NEO Preparedness and Response

Education and outreach practices are a critical component of the Sendai Framework and best practice can be drawn from various branches of the disaster risk arena. Two important aspects are:

- *Familiarity and cultural history*—using narratives that communities can relate to are effective methods to increase awareness towards risk. “Not in my lifetime” and “not in my backyard” attitudes towards HILP risks result in failures in the adoption of preparation measures. Using cultural narratives to demonstrate people’s relationship with risk is a powerful tool that enables vulnerable communities to embody their risk.
- *Repetition*—a one-off engagement is not enough to ensure resilient and prepared communities. For example, communities, organizations, and the public are often asked to repeat First Aid training on a 3-year basis to ensure skills remain fresh in their minds in case they are needed. The same applies for risk preparation training—one engagement with communities may not be enough for HILP risks to ensure sustained community preparedness. Therefore, sustained and progressive outreach strategies should be employed to build resilient communities.

## What is Next After the Scenario-Based Exercise?

The scenario utilized in the 7th Planetary Defense Conference is a cautionary tale. It indicates a need to prepare now for what might come in the future, because even with advance notice, time may be limited to prepare. This scenario also chose Europe for the impact. Europe could very likely cope with such a disaster, through the UCPM and other solidarity and support mechanisms within the EU, as well as with potential support from international partners. But could other areas in the world also cope with such disasters? And could response systems cope with a larger event in Europe or elsewhere? This scenario shows that planetary defense is a global endeavor.

Scenario-based exercises have been widely used in the biosecurity arena—for example, Johns Hopkins University’s *Event 201* (Centre for Health Security 2019) and the UK government’s *Operation Cygnus* (Public Health England 2017) simulations of pandemic coronavirus and influenza virus outbreaks, respectively. However, many of the recommendations and outcomes from such exercises were not adopted into preparation planning for these risks.

Future thinking is essential in insuring that governments and their partners are not “fighting the last war,” but rather, preparing for all potential future risks. Other methodologies for futures thinking can be utilized by communities such as horizon scanning and expert elicitation methods to further establish potential threat scenarios for NEO impacts. The constant engagement of the planetary defense community and disaster preparedness and response community is essential in order to keep the world safe from potential disasters due to NEO impact.

## Footnotes

1 United Nations Office for Outer Space Affairs, European Space Agency, European Commission Joint Research Centre, Centre for the Study of Existential Risk at the University of Cambridge, United Nations Institute for Training and Research, European Commission’s Directorate-General for European Civil Protection and Humanitarian Aid Operations, Federal Emergency Management Agency of the United States, and Hagerty Consulting.

2 The four panels included Disaster Managers Discussion, Reliable Communication and Dealing with Misinformation, Lessons Learned from Past Disasters: Recommendations for a Real NEO Threat, and The Planetary Society.
